# Volatile organic compounds influence prey composition in *Sarracenia* carnivorous plants

**DOI:** 10.1371/journal.pone.0277603

**Published:** 2023-04-19

**Authors:** Corentin Dupont, Bruno Buatois, Jean-Marie Bessiere, Claire Villemant, Tom Hattermann, Doris Gomez, Laurence Gaume

**Affiliations:** 1 AMAP, Montpellier University, CNRS, CIRAD, INRA, IRD, Montpellier, France; 2 CEFE, Montpellier University, CNRS, EPHE, IRD, Montpellier, France; 3 ENSCM, Montpellier University, Montpellier, France; 4 ISYEB, CNRS, MNHN, EPHE, Sorbonne University, Antilles University, Paris, France; Indian Institute of Science, INDIA

## Abstract

*Sarracenia* pitcher plants display interspecific differences in prey, so far only explained by pitcher morphology. We hypothesized that pitcher odours play a role in prey composition. We first compared odour and prey compositions among *Sarracenia* taxa grown together, forming a kinship gradient from *S*. *purpurea* known to capture primarily ants towards *S*. *leucophylla* known to capture many flying insects: *S*. *purpurea*, *S*. X *mitchelliana*, and *S*. X Juthatip soper & *S*. X *leucophylla* horticultural hybrids. We then measured several pitcher traits to disentangle the contributions of morphology and odour to prey variation. The pitcher odours were as diverse as those of generalist-pollinated flowers but with notable differences among taxa, reflecting their relatedness. VOC similarity analyses revealed taxon specificities, that mirrored those revealed by prey similarity analyses. *S*. X *leucophylla* stood out by being more specialised in flying insects like bees and moths and by releasing more monoterpenes known to attract flower visitors. *S*. X Juthatip soper trapped as many bees but fewer moths, sesquiterpenes contributing less to its scent. Ants and Diptera were the main prey of the other two with fatty-acid-derivative-dominated scents. Quantities of the different prey groups can be inferred 98% from quantities of the odour classes and pitcher dimensions. Two syndromes were revealed: ants associated with fatty-acid-derivatives and short pitchers; flying insects associated with monoterpenes, benzenoids and tall pitchers. In *S*. X *leucophylla*, emission rate of fatty-acid-derivatives and pitcher length explained most variation in ant captures; monoterpenes and pitcher length explained most variation in bee and moth captures; monoterpenes alone explained most variation in Diptera and wasp captures. Our results suggest that odours are key factors of the diet composition of pitcher plants. They support the hypothesis of perceptual exploitation of insect biases in carnivorous plants and provide new insights into the olfactory preferences of insect groups.

## Introduction

Plants are sedentary organisms and have developed over the course of evolution a particularly diverse and effective language for communicating at a distance with each other or with organisms from other kingdoms: odours with an alphabet made of volatile organic compounds [1,2]. Plants communicate with each other and use particular combinations of VOCs to warn their neighbours of herbivore attacks, or, conversely, to inhibit the germination of their competitors [3]. Plants also communicate with other organisms, by emitting repellent odours to deter pathogens [4,5] and herbivores [6,7] or by emitting COVs attractive to insects, which often perform essential functions in mutualistic relationships with them, such as defense against herbivores [8], pollination [9], seed dispersal [10], and even nutrition in carnivorous plants [11,12]. Carnivorous plants, to overcome the lack of nutrients in the soils where they grow, do indeed supplement their diet with essential nutrients obtained from insects and other arthropods that they attract, capture and digest with their highly-modified leaves [13–15]. Attraction is thus the first, but not least, component of the carnivorous syndrome in these specialised plants [13].

A group of carnivorous plants, the so-called pitcher plants, includes the well-known Sarraceniaceae from the Americas and the Nepenthaceae from Southeast Asia. The results of some studies comparing insect prey or inquiline numbers of pitcher traps *versus* control traps suggest that pitchers are not simple pitfall traps, highlighting the importance of attraction in these carnivorous plants [16,17]. Attraction in these pitcher plants is actually satisfied not only by olfactory signals [18–20] but also by other lures, such as nectar guides [21,22] and colour patterns [23–25]. Olfactory cues have received comparatively little attention. A few old studies used tissue extraction to investigate the odour of pitcher plants, but this method also collected non-volatile compounds [18,26] and thus did not provide information on the composition of the emitted bouquet. A more recent study used the same method to compare metabolites among Sarraceniaceae species and also investigated their scent profile but the investigation was then focused on the search for the volatile alkaloid coniine [27]. Only a few studies have actually examined the volatile compounds in the odour bouquets of *Sarracenia* [19,28] and *Nepenthes* [20] pitchers and have shown some similarity with flower scents. The role of pitcher scent in attracting insects has been put forward in *Sarracenia flava* and *S*. *leucophylla* where significant correlation between fly visits and VOC emission rates have been found [19] and has been demonstrated experimentally via olfactometry on ants and flies in *Nepenthes rafflesiana* [20] confirming earlier hypotheses [29,30].

Odours play an important role in the pollination systems of flower plants and the VOC composition of flowers sometimes characterises species-specific pollination systems or whole syndromes attracting a particular type of animals (e.g. bees in melittophile plants with an often terpinoid-rich cue; flies in myophile plants with odors often rich in aliphatics and nitrogen-compounds, butterflies in psychophile plants with a benzenoid-rich cue, bats in chiropterophile plants with sulfur-containing fetid odors) [31–33]. Thus, one may wonder whether in carnivorous pitcher plants, which show some inter-specific partitionning of prey [34], especially in those growing in sympatry where interspecific competition is high [35–37], odour plays a role in the differences observed in their prey spectra.

In *Nepenthes* pitcher plants, in addition to pitcher morphology and visual characteristics [23] as well as trapping mechanisms [38,39], odour is strongly suspected to participate in prey segregation (i.e. substantial variation in prey spectra) [35]. A large part of the differences in the amounts of flying and terrestrial insects trapped in pitchers can be explained by differences in VOC emission [20]. In *Sarracenia*, morphology has hitherto been mainly invoked to explain these differences in prey capture [36,40,41]. But Ellison and Gotelli reported only few differences in prey spectra among *Sarracenia* species. They even concluded that these carnivorous pitcher plants would act as passive traps [42], since the relative abundance of the different groups of prey arthropods trapped by the pitcher plants did not differ from the relative abundance of arthropods found in their environment. Their meta-analysis concluded that catches were random, which leaved little room for olfactory signals.

On the contrary, we hypothesise that olfactory signals play an important role in the prey composition of *Sarracenia* pitcher plants and that catches are not random. To test this hypothesis, we explored differences in odour emission and prey capture in four *Sarracenia* taxa grown together under the same biotic and abiotic conditions and differing by pitcher morphologies. The taxa, including two natural species and two horticultural hybrids formed a kinship gradient from *S*. *purpurea* (known to capture mainly ants [43,44] towards *S*. *leucophylla* (known to capture many flying insects [37]). We then investigated the extent to which the amounts of various classes of compounds emitted by pitchers, classified according to their biosynthetic pathways, explained the variations in captures of different insect groups.

## Material and methods

### Plant taxa and growing conditions

**Fig 1:**
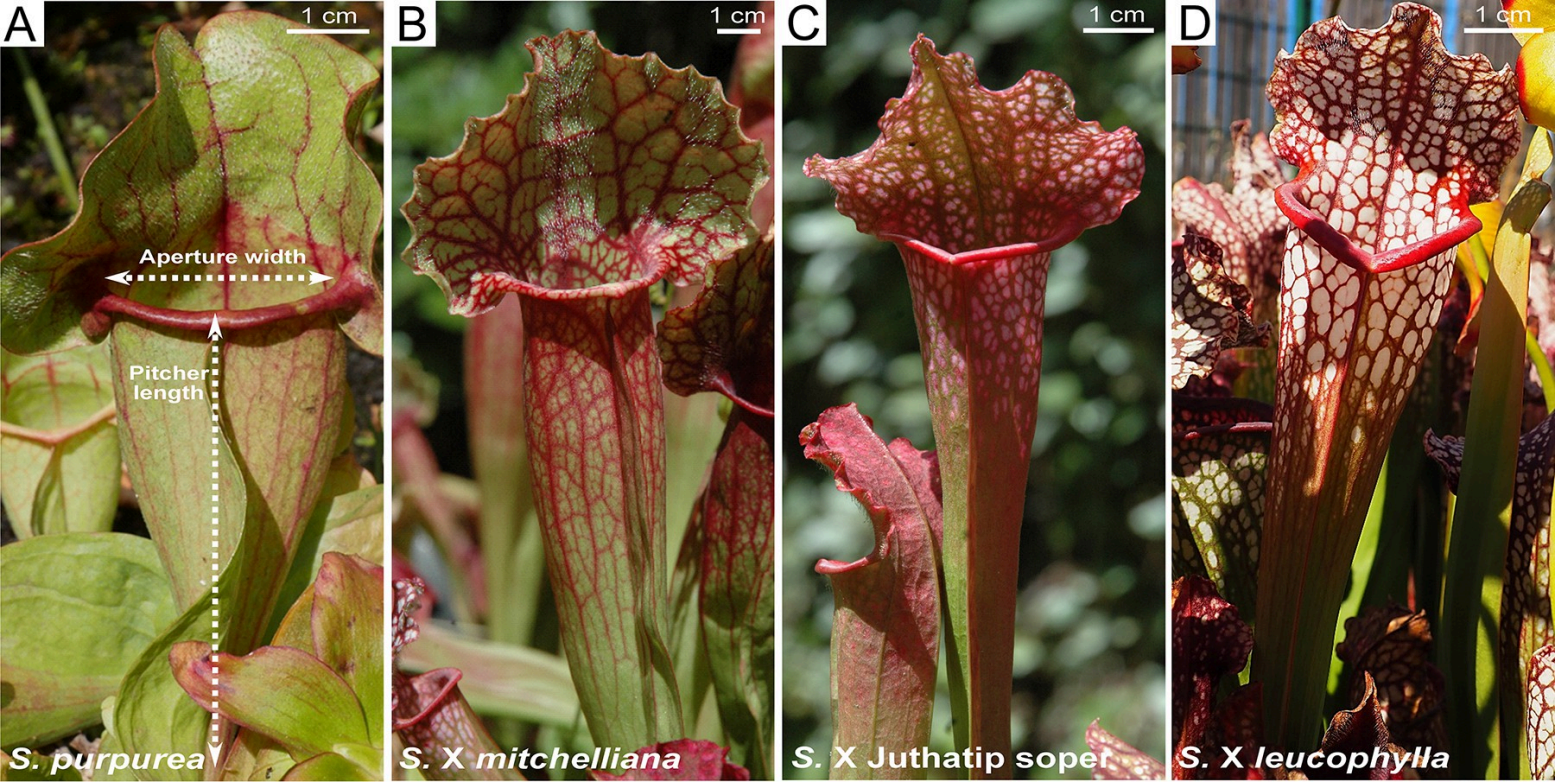
Pitchers of the four studied *Sarracenia* taxa. (A) *S*. *purpurea*, (B) *S*. X *mitchelliana*, (C) *S*. X Juthatip soper, (D) *S*. X *leucophylla*.

We considered four *Sarracenia* taxa differing in visual aspect and leaf shape (Fig 1). All the studied plants were horticultural specimens obtained from the carnivorous plant nursery “Nature and Paysages” (Peyrusse-Massas, Gers, France) at the same juvenile stage and replanted in the beginning of April 2016 in the experimental station of AMAP (Montpellier, France). Two of the taxa were natural species: *S*. *purpurea*, and *S*. X *mitchelliana* = *S*. *purpurea* X *S*. *leucophylla*, a natural hybrid from southern Alabama and northwestern Florida [45]. The two other taxa used in this study were horticultural hybrids: *S*. X Juthatip soper = *S*. *leucophylla X S*. *X mitchelliana* and *S*. X *leucophylla* = *S*. *leucophylla* X *S*. X Juthatip soper. Therefore, given the successive backcrosses with *S*. *leucophylla*, the four taxa follow a gradient of relatedness from *S*. *purpurea* towards *S*. *leucophylla*. *S*. *purpurea* has been divided into two main subspecies: the northern *purpurea* and the southern *venosa* subspecies [46]; the specimens we used belonged to the *venosa* subspecies. There is a controversy over the status of the *S*. *purpurea* taxon composing the *mitchelliana* hybrid, which in 2011 is still considered *S*. *purpurea* subsp. *venosa* var. *burkii* by Schnell who originally described it in 1993 [47], whereas in 1999 Naczi and co-authors elevated it to species status, as *S*. *rosea* [48]. Regardless of its status, this taxon is genetically closer to the *S*. *purpurea* taxa than to the other *Sarracenia* species, since according to all existing molecular phylogenies of the Sarraceniaceae and notably the most resolved one from Stephens et al. (2015) [46], it belongs to a common clade with only all other *S*. *purpurea* taxa. Furthermore, it is an intermediate taxon in this monophyletic group called *’purpurea* complex’ by Stephens and co-authors [46] framed by *S*. *purpurea* ssp. *venosa* var. *montana* and *S*. *purpurea* ssp. *venosa*. The nomenclature of Schnell classifying this taxon as a *S*. *purpurea* species and a variety of *venosa* subspecies is thus well supported.

The plants of the four taxa were grown outdoors in polyculture in the same 1m^3^ rectangular container (one row of plants per taxon). The container was filled with a peat mixture consisting of 2/3 blond peat and 1/3 sand on a background of clay balls in a sunny place. It was regularly watered with demineralized water to match their natural moist and mineral-poor environment. The study took place in the south of France during the summer of 2016, characterised by rather hot temperatures typical of the Mediterranean climate.

### Collection of volatile organic compounds

Plant odours were collected under natural light and ambient temperature by the dynamic headspace method and the adsorption-desorption technique. We collected the odours of a total of 39 pitchers on 16 different plants (7–13 pitchers per taxon see S1 Table) in 13 extraction sessions haphazardly carried out in the morning or afternoon of 7 days between 27/07/2016 and 25/08/2016. Pitchers were selected at comparable stages (15 ± 5 days after opening) for most of them or at a somehow wider range of stages centered on 15 days of opening when several were selected from the same plant so that stage means and variances were comparable among plant taxa. Stages were either directly measured or assessed based on multiple criteria of pitcher color, morphology and toughness established in a prior study where pitchers were monitored from opening date (unpublished results). Each studied pitcher was enclosed in a polyethylene terephthalate bag (Nalophane®, Kalle Nalo GmbH, Wursthüllen, Germany) tightly closed with cotton string. Airflow was maintained through the bags by two standard 12-V air pumps connected with silicone tubes to flowmeters. Pure air, filtered by charcoal filters, was flown into the bags at 60 mL/min and extracted at 40 mL/min through silicone tubing. The difference in flow made it possible to cope with the inevitable leaks and prevent outside air from entering the system. Pure air was flown into the bags for 10 min before starting dynamic extraction. The adsorbent traps attached to silicone tubing and put above pitcher aperture in the collection bags were ChromatoProbe® quartz microvials of Varian Inc. (length: 15 mm; inner diameter: 2 mm, previously cut closed-end) filled with 3 mg of a 1:1 mix of Tenax-TA and Carbotrap® (60–80 and 20–40 mesh, respectively; Sigma Aldrich, Munich, Germany). Empty bags with similar traps were used as ambient controls to check for possible contaminations during each set of VOC collections. ChromatoProbe® samples were stored at −20°C in a freezer until analysis, which was made within 1 month of collection. One microliter of a solution of two internal standards (n-Nonane and n-Dodecane, 108 and 114 ng/μL, respectively) was added to each ChromatoProbe® trap before scent extraction to ensure that samples did not suffer loss during collection and sample processing. Dose-response curves using standard compounds representing each class of VOCs found in the pitchers have also been processed to correct for the quantitative losses during sample processing. These two approaches allowed us to carry out reliable quantitative analyses and thereby comparisons of emission rates between pitchers.

### Chemical analysis

Samples were analysed with a gas chromatograph (GC, Trace™ 1310, Thermo Scientific™ Milan, Italy) coupled to a mass spectrometer (ISQ™ QD Single Quadrupole, Thermo Scientific™ Milan, Italy) and an Optima 5-MS capillary column (30 m, 0.25-mm internal diameter, 0.25-μm film thickness, Machery-Nagel, Düren, Germany). Absorbent traps were handled with a Multi-purpose sampler (Gerstell, Mülheim, Germany) and desorbed with a double stage desorption system, composed of a Thermal Desorption Unit (TDU) and a Cold Injection System (CIS) (Gerstell, Mülheim, Germany). First, filters were desorbed in splitless mode with a temperature of 250°C on the CIS trap cooled at −80°C by liquid nitrogen. Then, the CIS trap was heated to 250°C with a 1:4 split ratio to inject the compounds into the column. The carrier gas used was helium at 1 ml/min. Oven temperature was held at 40°C for 3 min, increased from 40 to 200°C at a rate of 5°C/min and from 200 to 250°C at 10°C/min, and finally held for 2 min. The temperature of the transfer line and the ion source of the mass spectrometer were 250 and 200°C, respectively. The acquisition was from 38 to 350 *m*/*z*, at a 70-eV ionization energy.

We used the standard retention times of C8 to C20 *n*-alkanes (Alkanes standard solution, 04070, Sigma Aldrich®) to convert retention times into retention indexes. We identified volatile organic compounds by comparing mass spectra with whose of database (NIST 2007 MS library, Wiley 9^th^ edition) and retention indexes reported in the literature [49]. Identifications were done using netCDF converted files on the GC-MS solution software (v4.11 SU1, Shimadzu Corporation, Kyoto, Japan). Peaks of identified VOCs were confirmed and integrated in each sample using Xcalibur® software (Thermo Scientifc^TM^ Milan, Italy). Compounds present in the controls were considered to be contaminants and their quantity was subtracted from each associated sample.

### Pitcher morphological measurements and prey capture analysis

22 pitchers out of the 39 analysed for their scents were also analysed for their prey contents and pitcher morphology. Thus, at the end of 4 days of odour sampling (corresponding to 7 sampling sessions) during the second half of August, a total of 22 pitchers of 11 plants (*S*. X *leucophylla* = 9, *S*. X Juthatip soper = 6, *S*. X *mitchelliana* = 3 and *S*. *purpurea* = 4) were cut and measured for pitcher length and aperture width (see Fig 1). Prey content was preserved in 70% ethanol and identified by Claire Villemant, at least to the family level for all taxonomic orders except Lepidoptera and Diptera, whose soft bodies were more digested preventing sometimes more precise identification. To obtain a more representative sample of pitcher mensurations and prey contents for each taxon, the 25^th^ of August 2016, we further carried out the measurements and prey identification of 19 other pitchers aged of 15 ± 5 days after opening and belonging to 18 plants (*S*. X *leucophylla* = 2, *S*. X Juthatip soper = 4, *S*. X *mitchelliana* = 7 and *S*. *purpurea* = 6). Unfortunately, it was not possible to sample the odours of these further pitchers.

### Statistical analysis

First, we compared pitcher scent and pitcher prey composition among the four different taxa. We therefore performed non-metric multidimensional scaling (NMDS) based on Bray–Curtis similarities of relative proportions (of VOCs or prey groups), using the “metaMDS” function from the R package “Vegan” [50]. The significance of differences in scent and prey patterns among taxa was assessed by permutational multivariate analyses of variance (PERMANOVA) with 999 random permutations using the “adonis2” function from the R package “Vegan” and pairwise comparisons using “pairwise.adonis2” function from the R package “pairwiseAdonis” [51]. P-values were adjusted for multiple comparisons using the Holm’s method. We also considered the variable ‘Plant identity’ nested in the variable ‘Plant taxon’ in our analyses.

Second, we used general linear mixed models to investigate wether the total emission rate and those of the different compound classes (benzenoids, fatty acid derivatives, monoterpenoids and sesquiterpenoids) varied according to the taxon considered. We analysed several dependent variables, i.e. the total emission rate (absolute quantity of VOCs), the emission rates of the four classes of compounds and the relative quantities of the four classes of compounds released by pitchers, with taxon as explanatory variable. As some of the pitchers belonged to the same plant and several samples were taken in the same sampling session, we considered the variables plant identity and sampling session as random variables. Absolute and relative quantities were square-root transformed to achieve normality of residuals.

Third, we used the same approach to investigate whether the total number of prey items and the number of prey items for different taxonomic groups varied according to the taxon considered. We thus analysed with general mixed models the total number of prey individuals trapped by pitchers, the number of flying prey individuals, the number of bees, moths, Diptera (flies, midges and mosquitoes), wasps (solitary, social and parasitoid wasps) and ants trapped by pitchers as dependent variables, testing taxon as an explanatory fixed variable and sampling day and plant identity as random variables. As the four taxa differed significantly in pitcher length (S1 Fig), which was likely to influence the number of prey [36,40,41], we corrected prey number by pitcher length in the analyses and considered this variable as a density (number of individuals / pitcher length). Density was square-root transformed to achieve normality of residuals.

Finally, we explored whether prey spectra can be explained by scent spectra in addition to pitcher morphology. We first investigated this for the four taxa using the pitchers for which we got both scent and measurement data, using a canonical correlation analysis (CCA) and the R package “CCA” [52]. This analysis aimed to explain the relationship between prey variables, i.e. the insect number of the main prey groups, and plant variables, which were the absolute VOC quantities of each compound class produced by pitchers as well as pitcher length and aperture width. Investigating further the correlations between plant taxa and prey variables observed in the CCA using general linear models implied testing too many taxon-variable interactions. Hence, we decided to focus on *S*. X *leucophylla* for which we had the highest sample size. Using multiple regression models with a Poisson distribution and a log-link function, we explored which variables–the absolute quantities of each compound classes and/or the morphological measures—best explained the variation in individual captures for each prey group (ants, Diptera, wasps, bees, moths and beetles) observed in this taxon. Since pitcher length and aperture width were highly correlated within the same taxon (e.g. *S*. X *leucophylla*, S4 Table) and, unlike length, width did not differ between taxa, we considered only pitcher length in these analyses for reasons of parcimony.

For all linear models, backward selection and Type III tests were carried out and we selected the best model with the lowest AIC (Akaïke criterium). Mixed models were estimated with the REML (Restricted Maximum Likelihood) method using the function “lmer” from the R package “lme4” [53]. Models were then adjusted with the ML (Maximum Likelihood) method and update function to get a better estimate of the coefficients for the fixed factors. For the last five Poisson multiple regression models only, a stepwise (forward-backward) selection of variables was preferred because it is a classical and appropriate procedure when several quantitative variables and a small sample size are involved. Indeed, by keeping fewer variables than the backward selection, it favours omission of variables that contributes little to the model, which is a favoured procedure because although it slightly biases the least square estimates, it also decreases the variance and mean square error of all least square estimates [54]. For each significant factor, post-hoc tests were carried out between any two factor levels, when necessary, using the function “contrast” from the R package “emmeans” [55]. P-values were adjusted for multiple comparisons using the Holm’s method. Statistics were performed using the software R version 4.0.3 [56]. Model assumptions were checked by plotting residuals against fitted values.

## Results

In summer 2016 in Montpellier, the pitchers of *Sarracenia* cultivated plants were found to release rich and complex odours, including a wide diversity of compounds ranging from 41 to 65 identified VOCs depending on taxon, with an average emission rate of 46 to 155 ng/h (S1 Table, n = 39 pitchers). The VOCS released belong to four classes of compounds, monoterpenes, sesquiterpenes, benzenoids and fatty acid derivatives (S1 Table). On the other hand, pitchers trapped up to 10 orders of animals, mostly insects (6 orders) with an average of 25±27 insect individuals per pitcher (n = 41), among which, hymenopterans were dominant (46%) followed by dipterans (26%), lepidopterans (11%) and coleopterans (9%). As the non-insect groups represented only 4 individuals they were not considered in the analyses.

### Interspecific variation in scent emission

**Fig 2:**
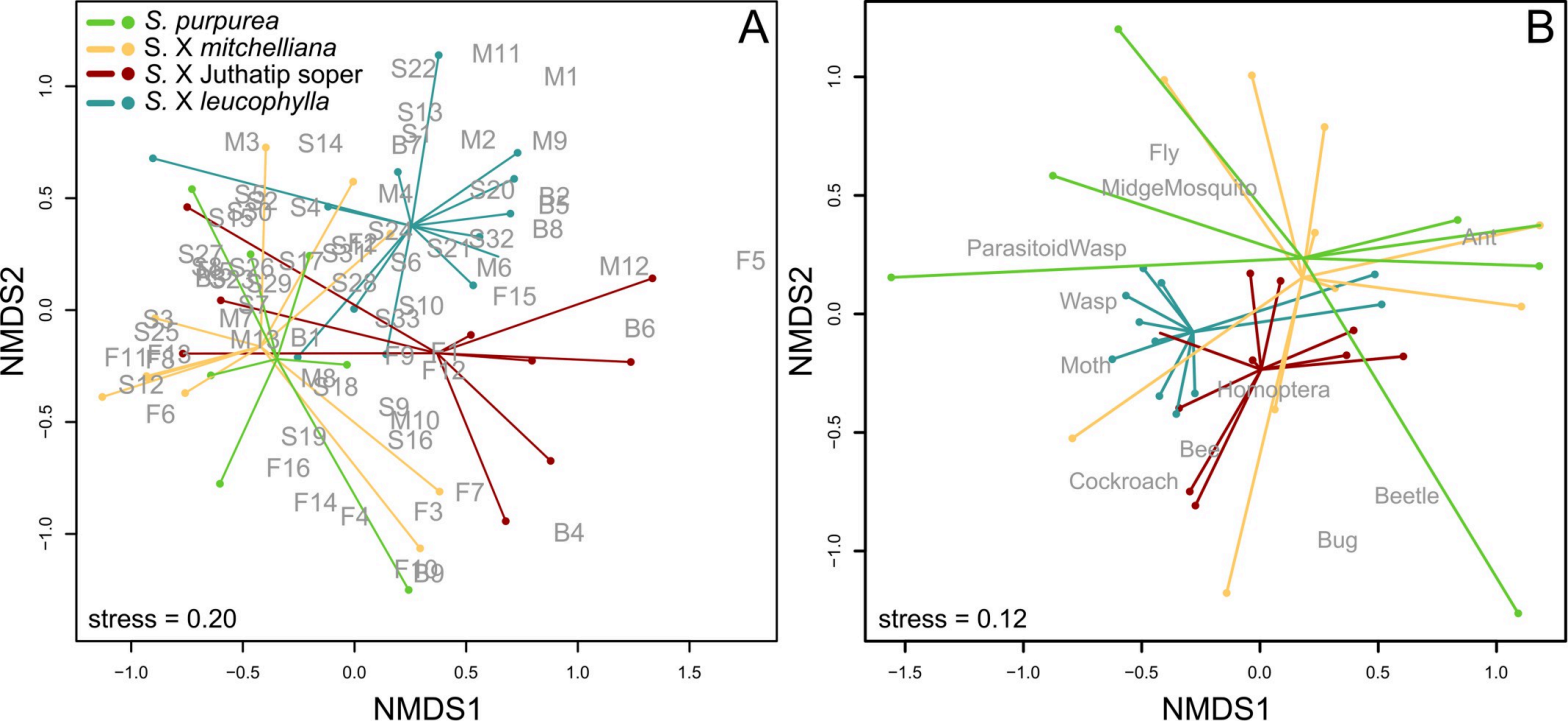
Similarity analyses of odour and prey composition of the *Sarracenia* taxa. Distribution of *Sarracenia* pitchers according to the similarity of their odour (A) and prey (B) spectra in terms of relative amounts of VOCs emitted and prey groups captured, respectively. The non-metric multi-dimensional scaling (NMDS) based on Bray-Curtis dissimilarity index showed a globally significant difference between *Sarracenia* taxa for both odour and prey spectra according to permutational multivariate analyses of variance considering also the effect of plant identity (P<0.001 in each case, see S2 Table for pairwise comparisons). The stress value was given to evaluate the quality of the ordination fit to the observed data. The VOCs are referred as Fi, Bi, Mi, Si, where i is the COV number and F, B, M and S refer to Fatty acid derivatives, Benzenoids, Monoterpenes and Sesquiterpenes respectively.

The pitchers of the same taxa grouped together based on the resemblance of their scent profile in terms of VOC composition (results of NMDS in Fig 2A) and the PERMANOVA (Table 1A) showed significant differences among taxa in the VOC profiles (Fig 2A). *Sarracenia* taxa thus differed globally in the odour bouquets of their pitchers (Fig 2A). However, there was a high intra-specific variability, shown by the sometimes-high distances in the scent profile between pitchers of the same taxon (Fig 2A), and also significant effects of “Plant identity” and “Sampling session” random variables in the analyses of compound-class emission. Therefore, only the odours of *S*. X *leucophylla* pitchers came out to be significantly different from those of the three other taxa (S2 Table).

**Table 1:** Result of similarity analyses of odour and prey composition of the *Sarracenia* taxa.

Dependent variable	(A) VOCs emitted			(B) Prey trapped		
Explanatory variables	F	df	P-value	F	df	P-value
Plant taxon	3.94	3	< 0.001 ***	4.90	3	< 0.001 ***
Plant identity	1.84	12	0.002 **	1.60	22	0.035 *

Factors of variation for the relative amounts of VOCs emitted (A) and prey trapped (B) by pitchers, from the results of the two PERMANOVAs.

*: P<0.05

**: P<0.01, and

***: P<0.001. Contrasts are detailed in S2 Table.

**Fig 3:**
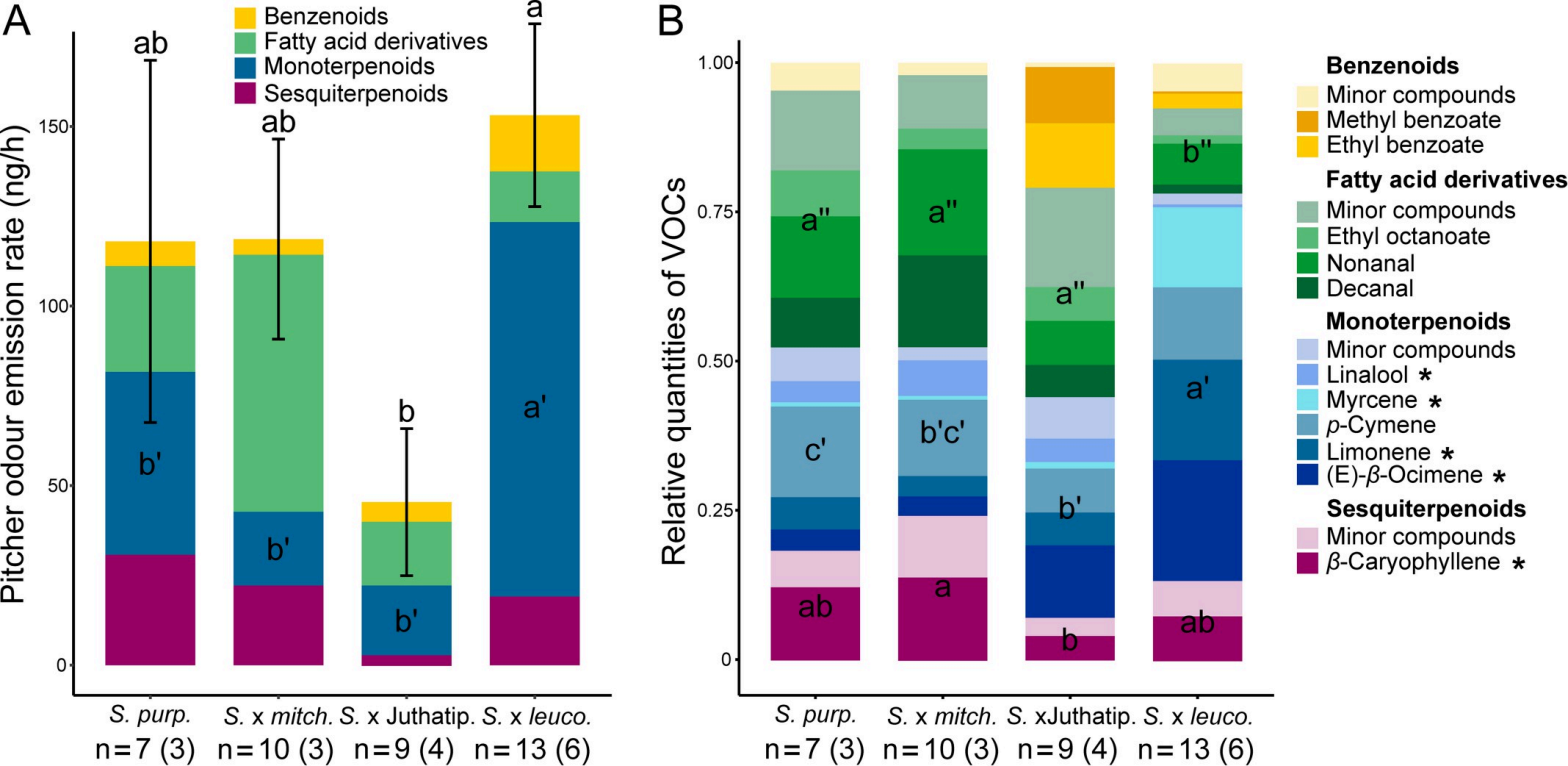
Odour profiles of the *Sarracenia* taxa. Odour profiles of pitchers of the four taxa in terms of emission rates (A) and VOC relative quantities (B). Mean values are presented with their associated standard errors (A). Only major VOCs representing more than 5% of the quantity of the odour bouquet were detailed (B). “*” indicates extremely common compounds in flower scents in Angiosperms (i.e. present in more than half of the seed plant families after Knudsen 2006). Different letters show statistically significant differences (P<0.05) in means between plant taxa for absolute (A) and relative (B) quantities of each class of VOCs according to the mixed linear models considering also the effects of the random variables “Sampling session” and ‘Plant identity” and using pairwise tests with Holm’s correction for multiple comparisons. The number of sampled pitchers is shown with the number of plants in brackets.

The odours of *S*. X Juthatip soper differed significantly from those of *S*. *X mitchelliana*. The odours of *S*. *X mitchelliana* revealed to be different from those of the two previous ones but not from those of *S*. *purpurea* (S2 Table). This highest singularity of *S*. X *leucophylla* regarding COV composition can be explained by the particularly high emission rate of monoterpenoids in this latter taxon (Fig 3A), which was significantly higher (P<0.01) than in the other three taxa, independently of any effects of sampling session and plant identity, which were considered in the model (Table 2). *S*. X *leucophylla* also stood out in terms of VOC diversity since up to 65 VOCs were found for this taxon, whereas there were no more than 56 VOCs identified in the other three taxa (S1 Table). The benzenoid class was particularly rich in this taxon, where twice as many compounds were found as in the other three taxa (S1 Table). In terms of emission rate, *Sarracenia* taxa did not vary significantly neither for benzenoids, nor for fatty acid derivatives, except weakly (P = 0.04) for sesquiterpenes, for which the emission rate was the lowest in *S*. X *Juthatip soper* (Fig 3A). Yet there were more marked interspecific differences in the relative amounts of the four classes of compounds. The share of benzenoids in the bouquet of *S*. X Juthatip soper, which had the lowest emission rate, was the highest but, statistically, it only came out to be higher than that of *S*. X *mitchelliana* with an almost significant trend (P = 0.05, Fig 3B). *S*. X *leucophylla* released a significantly lower proportion of fatty acid derivatives than the other three taxa and a significantly higher proportion of monoterpenes. *S*. X Juthatip soper displayed the second highest proportion of monoterpenes. Finally, the share of sesquiterpenes in the blend was the highest for *S*. X *mitchelliana* and the lowest for *S*. X Juthatip soper (Fig 3B). A total of 71 VOCs produced by the four *Sarracenia* taxa were identified (S1 Table). We observed some notable differences in the predominant compounds in the four plant taxa (Fig 3B). The bouquet of *S*. X *leucophylla* was clearly dominated by the monoterpenes, myrcene, (E)-*β*-ocimene and limonene; that of *S*. X Juthatip soper by (E)-*β*-ocimene and by the benzenoid esters, ethyl benzoate and methyl benzoate, that of *S*. X *mitchelliana* was dominated by the monoterpene, *p*-cymene, the sesquiterpene, *β*-caryophyllene and the two aldehydes, nonanal and decanal; while that of *S*. *purpurea* was dominated by *p*-cymene, *β*-caryophyllene and the fatty acid derivatives, decanal, nonanal and ethyl-octanoate.

**Table 2:** Interspecific differences in odour analyses.

Dependent variable	(A) Emission rate of VOC classes					(B) Relative quantities of VOC classes					
	Explanatory variables	Chisq	Df	Df resid.	P-value	Explanatory variables	Chisq	F	Df	Df resid.	P-value
**Total VOCs**	Plant taxon	13.53	3	33	0.0036 **						
	*Plant identity*										
**Benzenoids**	Plant taxon	5.04	3	33	0.1688	Plant taxon		2.72	3	35	0.0593
	*Plant identity*					-					
**Fatty acid**	Plant taxon	1.29	3	33	0.7307	Plant taxon	29.92		3	33	0.0093 **
**derivatives**	*Sampling* *session*					*Sampling* *session*					
**Monoterpenes**	Plant taxon	40.84	3	33	<0.001 ***	Plant taxon	41.18		3	33	<0.001 ***
	*Sampling* *session*					*Sampling* *session*					
**Sesquiterpenes**	Plant taxon	8.55	3	33	0.0359 *	Plant taxon	11.49		3	33	<0.001 ***
	*Plant identity*					*Plant identity*					

Factors of variation for (A) the emission rate (sqrt-transformed) of the odour bouquet and those of the different classes of VOCs composing the bouquet and (B) the relative quantities (sqrt-transformed) of each VOC class emitted. Random variables retained in models are presented in italics. “Chisq” values refer to type-III Walds tests for mixed models, “F” values refer to type-III F tests for non-mixed models performed when random variables were non-significant.

*: P<0.05

**: P<0.01, and

***: P<0.001.

### Interspecific variation in prey capture

**Fig 4:**
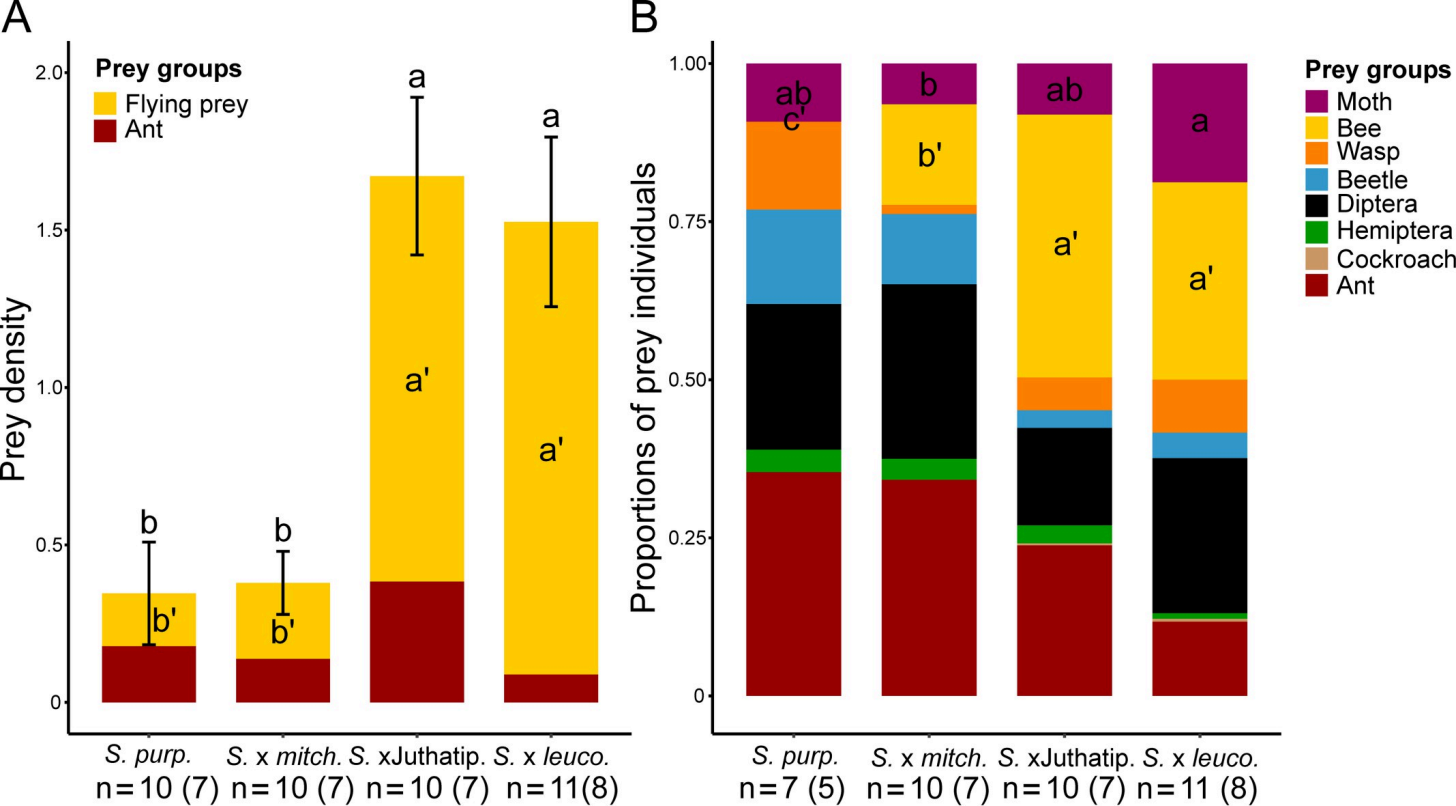
Prey profiles of the four *Sarracenia* taxa. Prey profiles of pitchers of the four taxa in terms of prey densities (Mean prey number/pitcher length ± SE, A) and relative numbers of individuals captured for each prey groups (B). Different letters show statistically significant differences in means between plant taxa for prey densities and proportions (P<0.05) according to the mixed linear models considering also the effects of the random variables “Sampling date” and ‘Plant identity” and using pairwise tests with Holm’s correction for multiple comparisons. Contrast for prey densities are detailed in S3 Table. Three pitchers of *S*. *purpurea* did not trap any prey and were therefore excluded from proportion analyses.

Pitchers from the same taxa were grouped together based on the resemblance of their prey spectra (Fig 2B) and the PERMANOVA showed significant differences among taxa in prey composition (Table 1B), with a structure of prey resemblance broadly similar to the structure of odour resemblance (Fig 2A and 2B, S2 Table). The prey compositions of *S*. X *leucophylla* and *S*. X Juthatip soper were significantly different from those of *S*. X *mitchelliana* and *S*. *purpurea* (S2 Table) and almost significantly different from each other (P = 0.06). *S*. *X purpurea* and *S*. *X mitchelliana*, which were grouped together, also both displayed higher intra-specific variance in their prey profile, compared to the other two taxa (Fig 2B, Table 1B). The total number of prey individuals significantly differed according to plant taxon even after correction by pitcher length (Fig 4A, Table 3A). *S*. X *leucophylla* and *S*. X Juthatip soper trapped a higher density of prey, especially of flying insects such as dipterans, moths and also bees than *S*. X *mitchelliana* and *S*. *purpurea* (Fig 4A, S3 Table). Bees trapped were mostly solitary bees with a clear dominance (92± 5%) of sweat bees (Halictidae). *S*. X *mitchelliana* trapped higher densities of bees than *S*. *purpurea* (S3 Table). Moths (especially Noctuidae *sensu lato*) were trapped in even greater densities in *S*. X *leucophylla* than in S. X Juthatip soper (S3 Table). The flies, which composed 84% of the dipterans and of which only 23% could be identified were, for the identified part, mostly composed of Phoridae detritivores (29%), Calliphoridae scavengers (18%), and Syrphidae pollinators (28%). The hoverflies (Syrphidae) were found almost exclusively in the pitchers of *S*. X *leucophylla* and *S*. X Juthatip soper. By contrast, the densities of ants and beetles did not differ significantly among the four taxa (Table 3A). In terms of proportions, the dominant prey groups also changed according to the taxon considered (Fig 4B, Table 3B). While ants and Diptera constituted the two most abundant groups in *S*. *purpurea* and *S*. X *mitchelliana*, they reached only 40% in *S*. X *leucophylla* and S. X Juthatip soper (with among these two groups, a dominance of Diptera in S. X *leucophylla* and a dominance of ants in S. X Juthatip soper), the remainder being composed mainly of bees (Fig 4B). The share of bees was significantly higher for S. X *leucophylla* and S. X Juthatip soper than the other two, *S*. *purpurea* did not trap any bees (Fig 4B, Table 3B). The share of moths was also the highest for *S*. *X leucophylla*, the lowest for *S*. X *mitchelliana* and intermediate for *S*. X Juthatip soper and *S*. *purpurea* (Fig 4B, Table 3B).

**Table 3:** Interspecific difference in prey captures.

Dependent variable	(A) Densities of prey groups					(B) Proportions of prey groups					
	Explanatory variables	Chisq	Df	Df resid.	P-value	Explanatory variables	Chisq	F	Df	Df resid.	P-value
**Total**	Plant taxon	61.55	3	35	<0.001 ***						
**prey**	*Sampling day*										
**Flying**	Plant taxon	101.58	3	35	<0.001 ***						
**prey**	*Sampling day*										
**Ants**	Plant taxon	6.31	3	35	0.0976	Plant taxon	3.22		3	32	0.3590
	*Sampling day*					*Sampling day*					
**Bees**	Plant taxon	191.77	3	35	<0.001 ***	Plant taxon		21.18	3	34	<0.001 ***
	*Sampling day*					*-*					
**Moths**	Plant taxon	51.86	3	35	<0.001 ***	Plant taxon	12.08		3	32	0.0071 **
	*Sampling day*					*Plant identity*					
**Diptera**	Plant taxon	33.12	3	35	<0.001 ***	Plant taxon	0.83		3	32	0.8429
	*Sampling day*					*Sampling day*					
**Wasps**	Plant taxon	21.58	3	35	<0.001 ***	Plant taxon	9.19		3	32	0.0269 *
	*Sampling day*					*Plant identity*					
**Beetles**	Plant taxon	2.22	3	35	0.5276	Plant taxon		0.50	3	34	0.6875
** **	*Plant identity*					*-*					

Factors of variation in (A) the density, i.e. number of individuals / pitcher length (sqrt-transformed) of the different insect groups trapped by pitchers and (B) the relative quantities (sqrt-transformed) of these groups of prey in terms of number of individuals. Random variables retained in models are presented in italics. “Chisq” values refer to type-III Wald tests for mixed models, “F” values refer to type-III F tests for non-mixed models performed when random variables were non-significant

*: P<0.05

**: P<0.01, and

***: P<0.001. Contrasts for prey densities are detailed in S3 Table.

### Correlations between odour, morphology and prey capture

**Fig 5:**
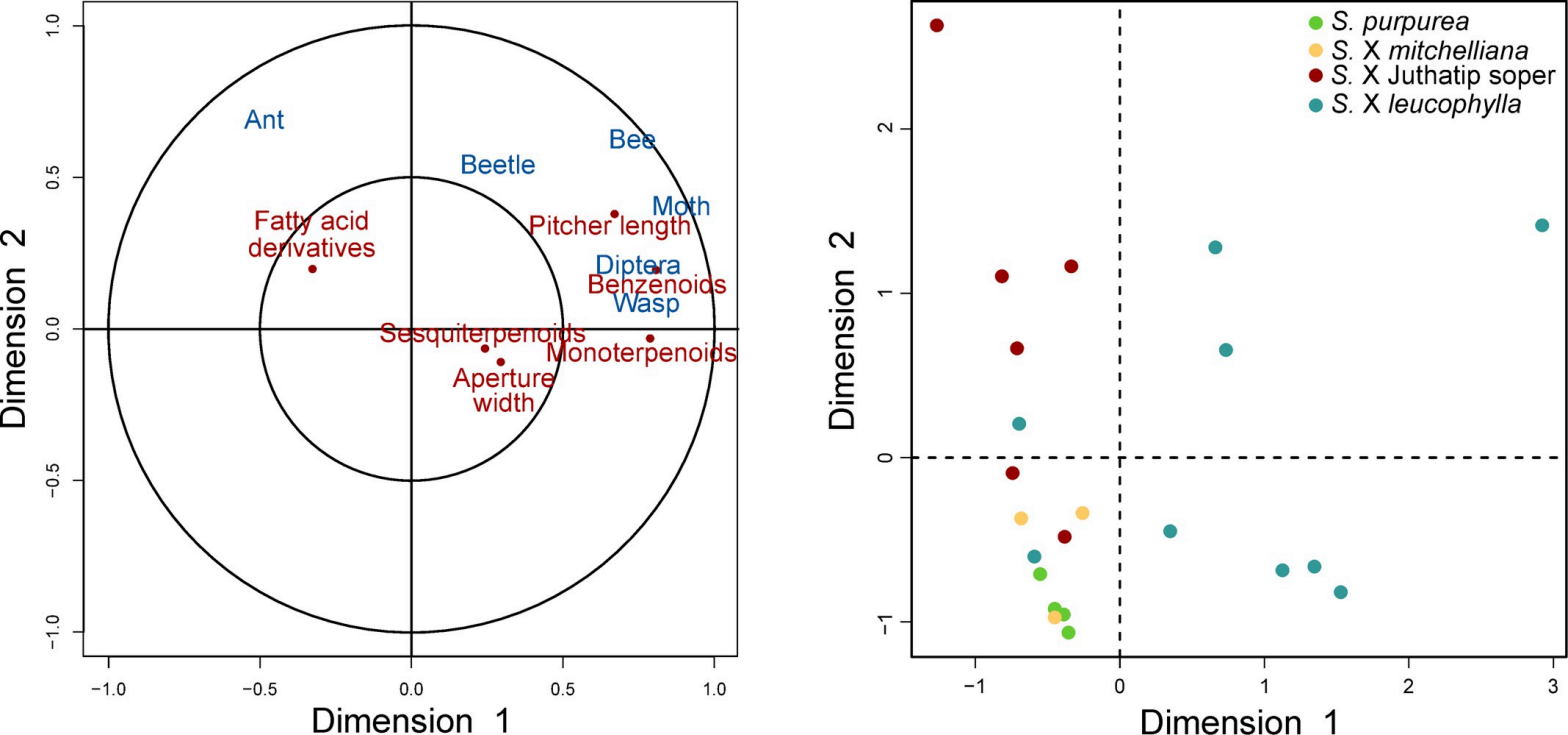
Canonical correlation analysis of plant and prey quantitative variables. Canonical correlation analysis between plant quantitative variables pertaining to odour & morphology and prey quantitative variables pertaining to the different insect groups trapped. (A) Representation of the plant (red) and prey (blue) variables on the correlation circle of the first two canonical axes. (B) Representation of the pitchers on the first two canonical axes.

According to the CCA (Fig 5A), the first two canonical correlations r1 = 0.98 and r2 = 0.76 were close to one meaning that plant and prey variables were highly correlated. There was a strong positive correlation of axis 1 with pitcher length, monoterpenoid and benzenoid emission rates on the one hand and with the number of flying prey (bees, moths, Diptera and wasps) on the other hand. Furthermore, axis 1 was negatively correlated with the number of ants trapped and the emission rate of fatty acid derivatives. Axis 1 opposed *S*. X *leucophylla* to the other three taxa (Fig 5B), this comparatively longer-leaf taxon being associated on the one hand with the release of higher amounts of monoterpenoids and benzenoids and fewer amounts of fatty acid derivatives, and on the other hand with more captures of flying insects and fewer captures of ants. Beetles, among flying insects, were an exception and contributed more to axis 2, which was also positively correlated, but to a lesser extent, with pitcher length and abundance of other prey since all the quantitative insect-variables were localised in the positive section delimited by this axis. Axis 2 separated *S*. X *mitchelliana* and *S*. *purpurea* shorter-leaved and less capture-efficient taxa from the two long-leaved *S*. X *leucophylla* and *S*. X Juthatip soper.

**Fig 6:**
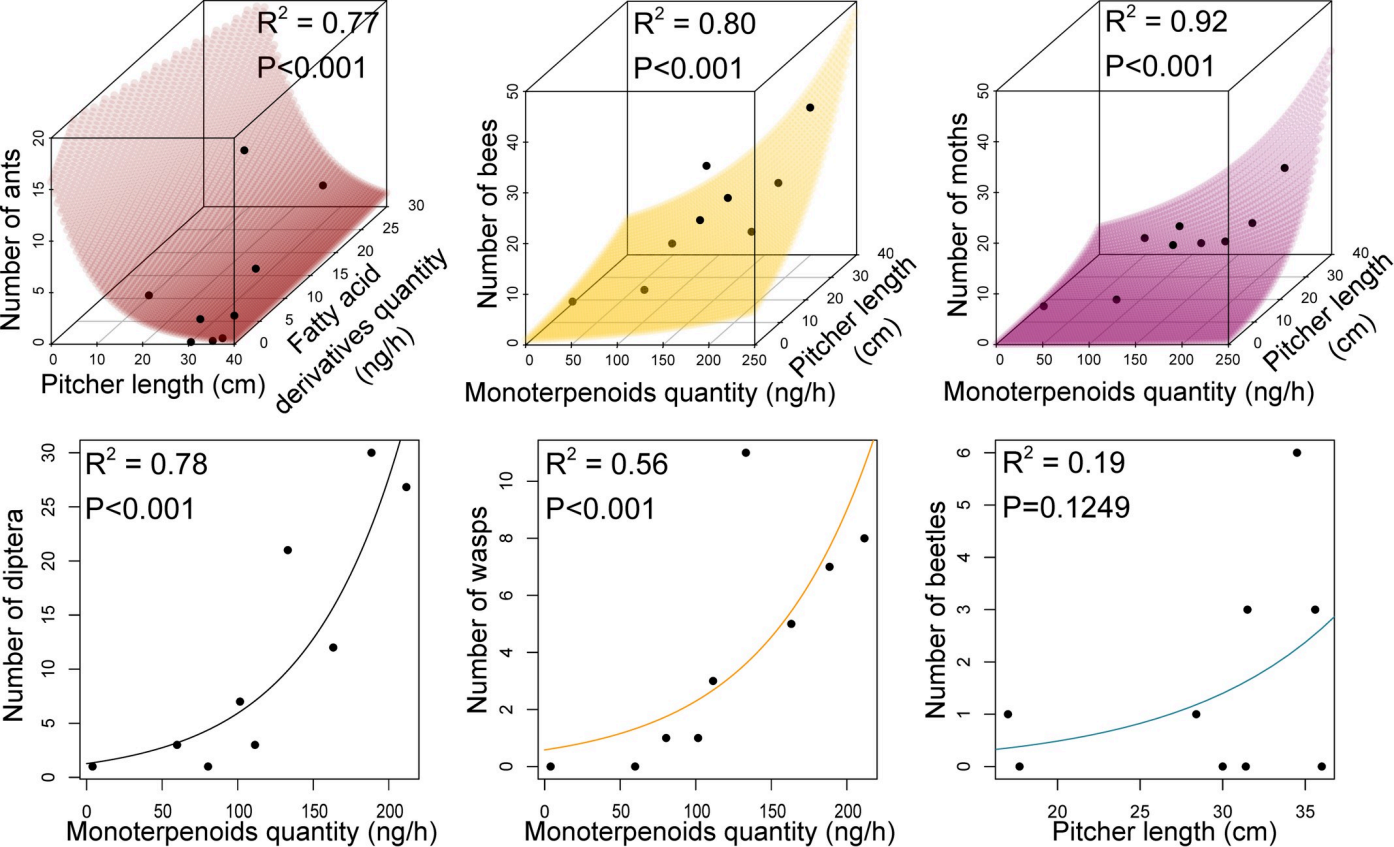
Relationships between prey capture and morphology & odour of *S*. X *leucophylla* pitchers. Relationships between prey numbers for the main groups of *S*. X *leucophylla* insect prey and the plant morphological and olfactory quantitative traits that were retained in the multiple Poisson regression models (Table 4). McFadden’s pseudo-R^2^ (1-(residual deviance/null deviance)) and overall significance of the models are indicated.

When considering *S*. X *leucophylla* only, we also found that both odour and pitcher morphology explained a high part of prey composition, and odour even more than pitcher morphology (Fig 6, Table 4). Regarding pitcher morphology, the number of ants trapped in pitchers decreased when pitcher length increased while the number of bees and moths increased when pitcher length increased (Fig 6, Table 4). As for odours, higher emission rates of fatty acid derivatives were associated with higher captures of ants while higher emission rates of monoterpenoids were associated with higher captures of bees, moths, Diptera and wasps (Fig 6, Table 4). The captures of Diptera and wasps were correlated only to olfactory traits and not to pitcher morphology. Moreover, while sesquiterpene and benzenoid emissions were highly correlated with bee and moth captures (S4 Table), they were not retained by the model because they explained less variation in the captures of bees and moths than did monoterpene emission and pitcher length variables to which they were also correlated (S4 Table). Conversely, beetle capture was not significantly dependent on either pitcher morphology or pitcher odour traits (Fig 6, Table 4).

**Table 4:** Effect of pitcher traits on captures of different groups of prey in *S*. *X leucophylla*.

Dependent	Explanatory	Estimate (±S.E.)	P-value	AIC	Null model AIC
variable	variables				
**Number**	Intercept	2.76 (±0.95)	0.0036 **	29.4	45.6
**of ants**	Pitcher length	-0.10 (±0.03)	0.0034 **		
** **	Fatty acid derivative quantity	0.05 (±0.02)	0.0232 *		
**Number**	Intercept	-0.83 (±0.77)	0.2804	55.0	101.1
**of bees**	Pitcher length	0.08 (±0.03)	0.0029 **		
** **	Monoterpene quantity	0.01 (±0.02)	<0.001 ***		
**Number**	Intercept	-2.27 (±1.16)	0.0502	38.8	69.7
**of moths**	Pitcher length	0.10 (±0.04)	0.0072 **		
** **	Monoterpene quantity	0.01 (±0.002)	0.0012 **		
**Number**	Intercept	0.24 (±0.35)	0.5000	58.3	127.8
**of Diptera**	Monoterpene quantity	0.02 (±0.002)	<0.001 ***		
**Number**	Intercept	-0.54 (±0.56)	0.3370	42.3	60.6
**of wasps**	Monoterpene quantity	0.01 (±0.003)	<0.001 ***		
**Number**	Intercept	-2.84 (±2.00)	0.1569	35.8	38.0
**of beetles**	Pitcher length	0.11 (±0.06)	0.0844		

Effects of the variables retained in the best Poisson multiple regression models to explain variation in the number of ants, bees, moths, Diptera, wasps and beetles trapped in pitchers of *S*. X *leucophylla*.

## Discussion

Our results based on a detailed study of the volatile organic compounds and prey individuals of some 40 pitchers of *S*. *purpurea*, *S*. *X mitchelliana* and further *S*. *leucophylla*-backcrossed hybrids show that these pitcher plants are quite generalist in their attraction systems and prey diet. However, despite high intraspecific variability, pitchers display taxon specificities in their scent and prey compositions, with similar inter-taxa differences in both odour and prey composition. We further show that the amounts in the blend of the different classes of volatile organic compounds, and to a lesser extent the length of the pitcher, explain the main differences observed in prey composition. These results show that catches are not random and suggest that odours contribute to control catches.

### A generalist scent signal with notable interspecific differences

The studied *Sarracenia* pitcher-shaped leaves release an odour rich in VOCs with a mean emission rate of 46 to 155 ng/h depending on taxon, which is in the range of flower emission rates [1,57]. Odours released by leaves usually have much narrower spectra and lower emission rates [58,59]. The bouquets released by the four *Sarracenia* taxa were composed of fatty acid derivatives, benzenoids and terpenoids. Such a composition is more typical of blends of generalist-pollination systems [1,33,58], and characterizes also the traps of other carnivorous plants [11,20]. Having a generalist olfactory signal is certainly advantageous for pitcher plants, which strongly rely on arthropods to obtain the nutrients indispensable to their growth [60]. A generalist scent is expected to increase prey diversification, otherwise the carnivorous plants could experience periods of starvation due to seasonality effects in the availability of specific insects.

In spite of this overall generalist character, some more specific syndromes seem to characterise the scents of the different taxa. *S*. X *leucophylla*, certainly displays the most flower-like scent of all taxa, because of the intensity of its perfume, and the release in high quantities of very common floral compounds. The monoterpenes, myrcene, (E)- *β*-ocimene and limonene, which dominated their bouquet, are present in the flower scents of two-thirds of the Angiosperm families [1]. They are often found in flower scents pollinated by bees and/or lepidopterans [31,33], which actually constituted half of the catches of *S*. X *leucophylla*. High emission rates of myrcene, a powerful attractant of Noctuidae *s*.*l*. [61], the predominant group of lepidopteran prey in these *Sarracenia* pitchers, are even a specificity of this taxon. In their study of *S*. *leucophylla* native species, Jürgens also described a floral scent with abundant monoterpenes but with higher quantity of benzenoids than found in this study [19]. In the field, *S*. *leucophylla*, being genetically close to *S*. *X leucophylla* backcrossed three times with *S*. *leucophylla*, was reported to trap mostly flying insects of large sizes including bees [36], sometimes with a dominance of flies [37], results which are consistent with our own observations on *S*. X *leucophylla*. It is thus probably a striking example, like *Nepenthes rafflesiana* in Southeastern Asia, of carnivorous plants where pitcher-shaped leaves biochemically “mimic” flowers [20], or at least exploit the sensory biases of generalist insect-pollinators [62]. By contrast, the scents of both *S*. *purpurea* and *S*. X *mitchelliana*, quite similar to each other, were dominated by fatty acid derivatives, and presented in their odour bouquet some characteristics of the myophile syndrome [33]. Their fatty acid derivatives are mainly the two aldehydes, nonanal and decanal, as well as alkanes and alkenes. While nonanal can effectively be associated with the attraction of carrion flies [63], nonanal and decanal (the latter with a more citrus-smell), are most often herbivore-induced plant volatiles known to attract insect predators [64] or host-seeking parasitoid wasps [65]. Predators and parasitoid compose a significant part of the prey in *S*. *purpurea* and *S*. X *mitchelliana* among ants, wasps and beetles. Alkanes and alkenes, which might also come from insects themselves and not exclusively from the plants, are also largely used in chemical communication in ants [66,67], some alkanes even known to trigger recruitment behaviour [66] in ants being the major prey group of these two plant taxa. Most previous studies on prey capture in *S*. *purpurea* also report a dominance of ants [21,43,44] except some, which report a dominance of flies [68,69]. The scents of *S*. X *mitchelliana* and *S*. *purpurea* also tend to have a greater proportion of sesquiterpenes than the other two plant taxa in their odour bouquets with a predominance of *p*-cymene and *β*-caryophyllene. These two terpenes are known to attract, in addition to some bees or moths [1], parasitoid wasps in response to herbivore attacks [70,71]. *β*-caryophyllene is also a common sesquiterpene with a spicy note that, when associated with fatty acid derivatives, attracts saprophagous and/or carrion flies [33], two also abundant groups of flies found in the pitchers of these two taxa. Furthermore, *p*-cymene and *β*-caryophyllene were two compounds particularly abundant in starving plants of the Venus flytrap [11], which as its name suggests captures mainly flies. As for *S*. X Juthatip soper, this taxon presents an olfactory syndrome halfway between the generalist-flower syndrome of *S*. X *leucophylla* and the fly & ant syndrome of *S*. *purpurea* and *S*. X *mitchelliana*. Indeed, its odour is less strong than that of *S*. X *leucophylla* and thereby less typical of flowers, but it keeps important shares of benzenoids, monoterpenes and fatty acid derivatives, which could still contribute to the attraction of flower visitors such as bees and moths, in addition to ants and Diptera. However, two dominant benzenoids in this taxa were methyl benzoate and ethyl benzoate, two compounds more commonly found in fermented fruits and known to attract fruit flies such as Drosophilidae [72] or Tephritidae [73], making its odour thus more fruity than floral.

### Prey variability globally mirrors scent variability at both intra and interspecific levels

The NMDS analysis shows that pitcher scents from the same taxon are overall more similar than pitcher scents from different taxa. However, it also illustrates a rather high variability in scent composition within the same taxon. In their meta-analysis, Delle-Vedove *et al*. highlighted the importance of intraspecific variation in floral scent in Angiosperms [74], where individual genetics, flower ontogeny, climatic parameters, interactions with insects are factors that can affect floral scent. Similar factors may influence pitcher scent. Part of the observed “within” variation might indeed reflect differences among individuals or among days or timings of odour sampling, as suggested by our analyses, which often retained the random variables "Plant identity" and "Sampling session" in the models on the relative amounts of the different classes of compounds. For example, the influence of temperature and humidity on monoterpene emission [75] might explain the effect of the session of scent sampling on this compound class. The observed intra-specific variation may also reflect a variation linked to pitcher age or to pitcher physiology. Indeed, the traps are likely to release their odour only when they need to feed and therefore reduce and/or change it after substantial captures, during the digestive process. Supporting this, is the report of a reduction of terpene emission in fed-traps compared to unfed-traps in an experiment carried out on the famous Venus fly-trap, Dionaea [11].

Furthermore, the NMDS graphs also show that the interspecific variation in prey composition roughly reflects that in scents. Indeed, in both ordinations (i.e. based on scent and prey profiles), the centroids of *S*. X *purpurea* and *S*. X *mitchelliana* are almost superposed, and those of *S*. X *leucophylla* and *S*. X Juthatip soper are closer to each other than to the other two. While *S*. X *purpurea* and *S*. X *mitchelliana* are closer to fatty acid derivatives and sesquiterpenes on the one hand, and closer to ants and flies on the other hand, *S*. X *leucophylla* and *S*. X Juthatip soper are closer to benzenoid and monoterpene compounds on the one hand and closer to flower visitors on the other. Interestingly, the distribution of taxa according to their odour and prey profiles also reflects their degrees of relatedness, including similar structures of odour and prey dissimilarities among taxa (Fig 2). This supports both the hypothesis of a genetic control of their odour patterns or at least their biosynthetic pathways and the hypothesis that odour ‘selects’ for prey. In other words, the horticultural hybrids would share the olfactory characteristics of their parents and the pitcher plants would exploit the sensory biases of insect groups in the same manner as their native counterparts and/or closest relatives, the sensory characteristics of which would have originally evolved to adapt to the ecological constraints and prey availability of their native environments.

At the intra-specific level, the highest variability in NMDS prey profile observed in *S*. *purpurea* and *S*. X *mitchelliana* is somehow also observed in the NMDS scent profile, suggesting that prey differences also mirror odour differences at the intraspecific level, but the variability is even higher for prey in these two taxa. High unevenness in prey capture was also reported in the field for *S*. *purpurea* [68]. An explanation, in addition to the scent-variability hypothesis, would be that capture is maybe less efficient in the shorter-leaved *S*. *purpurea* and *S*. X *mitchelliana* than in *S*. X *leucophylla* and *S*. X Juthatip soper, that would conversely capture more efficiently the insects which they attract. This latter hypothesis is supported by two reports. Newell and Nastase reported high escape rates in the short-leaved *S*. *purpurea* [43], while on the contrary, in the native and long-leaved *S*. *leucophylla*, the escape rates were very low [76]. The strong correlation between the quantities of compound classes and prey groups that we find for the longest-leaved *S*. X *leucophylla* further argue for a stronger link between prey and odour for pitchers of longer sizes. It also highlights the existence in carnivorous plants of different traits linked to attraction and trapping that play in concert to form trapping syndromes, as proposed by Juniper et al. [13] and further evidenced in *Nepenthes* genus [35].

### An overlooked but potentially important role of odours in the diet composition of pitcher plants

Of the signals involved in communication, odour is probably the most cryptic to humans, who primarily use their visual sense [77] and it may thus have been largely overlooked. In addition, odour is often correlated with other plant characteristics, making it difficult to disentangle its effect from others. Furthermore, it cannot be precisely described without a technological tool, i.e. the GC-MS. Its effect may also be not easily pointed out because different compounds that form the blend can have opposite effects on different guilds of insects. *Sarracenia* plants are not spared from these issues. The positive correlation revealed in this study between certain odour components such as the amounts of benzenoids and terpenes with pitcher length (S4 Table) could explain why the effect of odour could have remained unnoticed. Indeed, pitcher morphology, a set of traits easier to measure, was often shown to be “the” driver of prey abundance. Thus, some authors have already shown a correlation between pitcher length and prey amount in different *Sarracenia* species (Cresswell [70], Heard [44] in *S*. *purpurea*, Bhattarai and Horner [16] Green and Horner [78] in *Sarracenia alata* and Sheridan [41] in *S*. *leucophylla*). However, only Bhattarai and Horner obtained data, which let them suppose that this positive correlation was also due to “a greater quantity of attractants produced by larger pitchers”. Yet these authors did not test for any difference linked to insect guilds and/or taxonomy. Only Gibson [36] reported in *S*. *leucophylla* a positive correlation between pitcher size and prey number only for flying insects, which corroborates our own results.

An experimental approach involving olfactory tests is needed to get definitive proofs that odours are initial drivers of prey composition. Moreover, we cannot rule out the hypothesis according to which the observed positive associations between odour and prey patterns might be an artefact resulting from the process of human hybridization of two of these taxa. However, a number of arguments does not support the hypothesis that these patterns were observed by chance. First, the observed odour profiles seem to roughly reflect the genetics of these plants, chosen because they formed a gradient of relatedness from *S*. *purpurea* towards *S*. *leucophylla*. Indeed, their odours concomitantly show an increasing gradient in the relative amount of monoterpenes commonly found in flowers and a decreasing gradient in the relative amount of fatty acid derivatives. Second, it was found that the natural species *S*. *purpurea* and *S*. *leucophylla* emit mainly fatty acid derivatives for the former and floral compounds for the latter [19], exactly as we find for *S*. *purpurea* and *S*. *X leucophylla*, the taxon the most related to *S*. *leucophylla*. Next, we similarly find that ants are the main prey of *S*. *purpurea* as observed in its native environment and that flying insects are the main prey of *S*. *X leucophylla*, exactly like its most closely related natural species, *S*. *leucophylla* [37,76]. Moreover, the high strength of the observed correlations does not reflect the intervention of chance either. The canonical correlation analysis carried out on the four taxa shows that quantities of the different prey groups can be inferred 98% from quantities of the odour classes and dimensions of their pitchers. Taking pitcher size into account, the CCA illustrates how the number of ants is then positively correlated with the quantity of fatty acid derivatives and negatively correlated with the quantity of monoterpenes and benzenoids. Conversely, the number of flying insects is positively correlated with the quantity of monoterpenes and benzenoids. Moreover, this analysis shows a spatial separation of the taxa according to the first two canonical axes. The two axes separate from each other *S*. X *leucophylla*, *S*. X Juthatip soper, and the remaining couple, *S*. *purpurea* and *S*. X *mitchelliana*. These results are confirmed at the intra-specific level in *S*. X *leucophylla*, for which these odour components explain systematically a higher part of the variation in prey captures than does pitcher size, for each prey group separately. They also confirm the correlation between ant number and fatty acid derivative quantity, and show that the quantity of monoterpenes, among those of benzenoids and sesquiterpenes, which are mutually correlated, is the key odour component explaining variation of the different groups of flying prey tested except beetles, for which no significant correlation is found. While the negative correlation with pitcher size of ants, ground-dwelling insects, is easier to understand, the positive correlation of flying insects may be explained either by a greater attraction because taller pitchers are probably more conspicuous to flying insects, or by a greater retention of flying insects in taller and usually slender pitchers (personal observations CD, DG, LG, TH).

The positive correlation of ants with fatty acid derivatives is not surprising since ants use some of them in their chemical communication for nestmate recognition [67] or as pheromones [66] and since they are often released by plants, which attract ants as bodyguards [79]. Interestingly, the lower pitchers of the vine *Nepenthes rafflesiana*, which trap mostly ants, release greater percentages of fatty acid derivatives than the upper pitchers, which target flying insects, mostly flower visitors, with a blend rich in benzenoids and monoterpenes thus showing a compelling similarity with *S*. X *leucophylla* [20]. Bees and moths, important pollinators, are indeed known to be attracted by monoterpenes and benzenoids, which are typical flower components [31,33,80]. Ants constitute only a small proportion (12% ±7) of the prey in *S*. X *leucophylla* and their abundance turns to be negatively correlated with the quantity of monoterpenes in the CCA analysis including all taxa. Whether this is because the share of fatty acid derivatives is significantly lower in the scent of this taxon or because ants are repelled by a high abundance of typical flower compounds such as limonene (the second most abundant compound in this taxon) as suggested by an earlier study [81] remains to be clarified. Gibson reports that the native *S*. *leucophylla* captures some small insects, but collectively these contribute little to the total catch biomass, to which many larger insects contribute much more [36,76]. This suggests that ants constitute also a small portion of its prey. Resource partitioning between *Sarracenia* species is likely to occur in the bogs of southeastern USA where competition has been shown to be high between *S*. *leucophylla* and other *Sarracenia* species [36]. However, Ellison and Gotelli did not find strong evidence of resource partitioning among five *Sarracenia* species living in sympatry because they turned to trap mostly ants [42]. But in the *Sarracenia* community considered, several long-leaved species were absent, in particular *S*. *alata*, described by Gibson to produce “attractive odours” and to be found in sympatry with *S*. *leucophylla* in competitive relationships for flying insects. Indeed, ants are ubiquitous and may thus constitute a rather reliable source of food for pitcher plants, but the carnivorous plants are more likely to compete for bigger prey, which are more nutritive but also rarer, as suggested by Darwin [82] and pointed out by Gibson [36]. Analysis of the prey composition and odour pattern of these species would certainly help to better understand the evolutionary ecology of these carnivorous plants.

The question as to why *S*. X Juthatip soper, which emits fewer VOCs than *S*. X *leucophylla*, still attract as many flying insects, especially bees as *S*. X *leucophylla*, warrants further exploration. The stronger share of fatty acid derivatives in its blend associated to an equivalent share in benzenoids than *S*. X *leucophylla* could be an explanation. Indeed, Kantsa *et al*. in their meta-analysis reported the importance of the percentage of aliphatic for bee attraction [83]. But something else must also be at play. Other traits like colour signal is also an important aspect of attraction in the trapping syndrome of carnivorous plants [13]. In particular, pitcher red coloration has been proposed to play a role in prey attraction in pitcher plants [24,43]. However, this hypothesis has been refuted by some authors [21,41] arguing that it is not easy to disentangle the contribution of the different features since they are often combined to form whole trapping syndromes, with many traits targeting specific guilds of animals or characterising specific diets [35,84].

In conclusion, our study shows that the odours of *Sarracenia* pitchers are rich in VOCs, combining compounds from three biosynthetic pathways, with the pitchers of *S*. *X mitchelliana* and *S*. *purpurea* having an odour dominated by fatty acid derivatives, and the pitchers of the two taxa genetically closest to *S*. *leucophylla* having the most diverse and floral odours. This confirms the trends observed in the natural *S*. *purpurea* on the one hand and *S*. *leucophylla* on the other hand. Our study further brings together a set of presumptions that, in addition to pitcher morphology, olfactory cues play an important role in the diet composition of *Sarracenia* pitcher plants with monoterpenes and benzenoids associated with flying insects, and fatty acid derivatives more specifically associated with ants. Such trends observed in a non-native environment on *S*. *purpurea* and *S*. *leucophylla* backcrossed hybrids, which confirm the insect trends observed on *S*. *purpurea* and *S*. *leucophylla* native species in their own environments, provide support to the perceptual exploitation hypothesis [85]. Carnivorous plants would thus exploit insect sensory biases, i.e. general sensory preferences related to insect guild and/or taxonomy, a hypothesis, which contrary to what mimicry *stricto sensu* implies, does not rely on consistent spatio-temporal association between species [86]. Experimental studies on native species in their own environment and integrating different traits together, including visual ones, would certainly clarify and complement the different trapping syndromes of these carnivorous plants and shed light on the eco-evolutionary mechanisms underlying them.

## Supporting information

S1 Fig Variation of morphology among taxa. Pitcher length (A) and aperture width (B) in the four *Sarracenia* taxa. Mean values are presented with their associated standard errors. Different letters show statistically significant differences in means between plant taxa (P<0.05).
(TIF)

S1 Table VOC composition of *Sarracenia* taxa. Mean relative amounts (% ± SE) of VOCs in the odour bouquet of the four *Sarracenia* taxa. VOCs are listed according to their class and calculated Linear Retention Indices (LRI). Mass fragments for unknown compounds were listed with the molecular ion first, followed by the base peak and other fragments in order of decreasing abundance. IA: Identification Attempt, *: Identified with comparison with synthetic standards. Occurrence (Occ.) refers to the proportion of pitchers in which the VOC was found.
(PDF)

S2 Table Post-hoc tests of similarity analyses in odour and prey composition of the different *Sarracenia* taxa. Results of the pairwise comparisons between taxa associated to the permutational multivariate analyses of variance on the relative amounts of VOCs emitted (A) and prey group trapped (B) by pitchers (Fig 1). P-values were adjusted for multiple comparisons with the Holm’s correction, *: P<0.05, **: P<0.01, and ***: P<0.001. The plant effect (‘Plant identity’ nested within ‘Plant taxon’) was also accounted for.
(PDF)

S3 Table Post-hoc tests for prey density analyses according to taxa. Results of the pairwise comparisons between taxa associated to the models carried out to explain the variation in the sqrt-transformed density (number of individuals / pitcher length) of the different insect groups trapped by pitchers. Contrasts were tested between plant taxa using pairwise tests. P-values were adjusted for multiple comparisons with the Holm’s correction.
(PDF)

S4 Table Correlation between odour, morphology and prey capture parameters in *S*. X *leucophylla*. Correlation table relative to odour variables (emission rate of fatty acid derivatives, benzenoids, monoterpenoids and sesquiterpenoids), morphology variables (pitcher length and aperture width) and number of ants, bees, moths, Diptera, wasps and beetles trapped in pitchers of *S*. X *leucophylla*. The correlation coefficients between each pair of variables estimated using the Pearson method, are presented with their associated P-values, *: P<0.05, **: P<0.01, and ***: P<0.001.
(PDF)

S5 Table Effect of pitcher traits on prey-group captures in *S*. X *leucophylla*: Second-best model. Effects of the variables retained in the second-best Poisson multiple regression model to explain variation in the number of ants, bees, moths, Diptera, wasps and beetles trapped in pitchers of *S*. X *leucophylla*.
(PDF)
