# Supplementary figures and images for: Volatile organic compounds influence prey composition in Sarracenia carnivorous plants
Source: PLoS One. 2023 Apr 19;18(4):e0277603. doi: 10.1371/journal.pone.0277603 (PMC10115284; doi:10.1371/journal.pone.0277603)

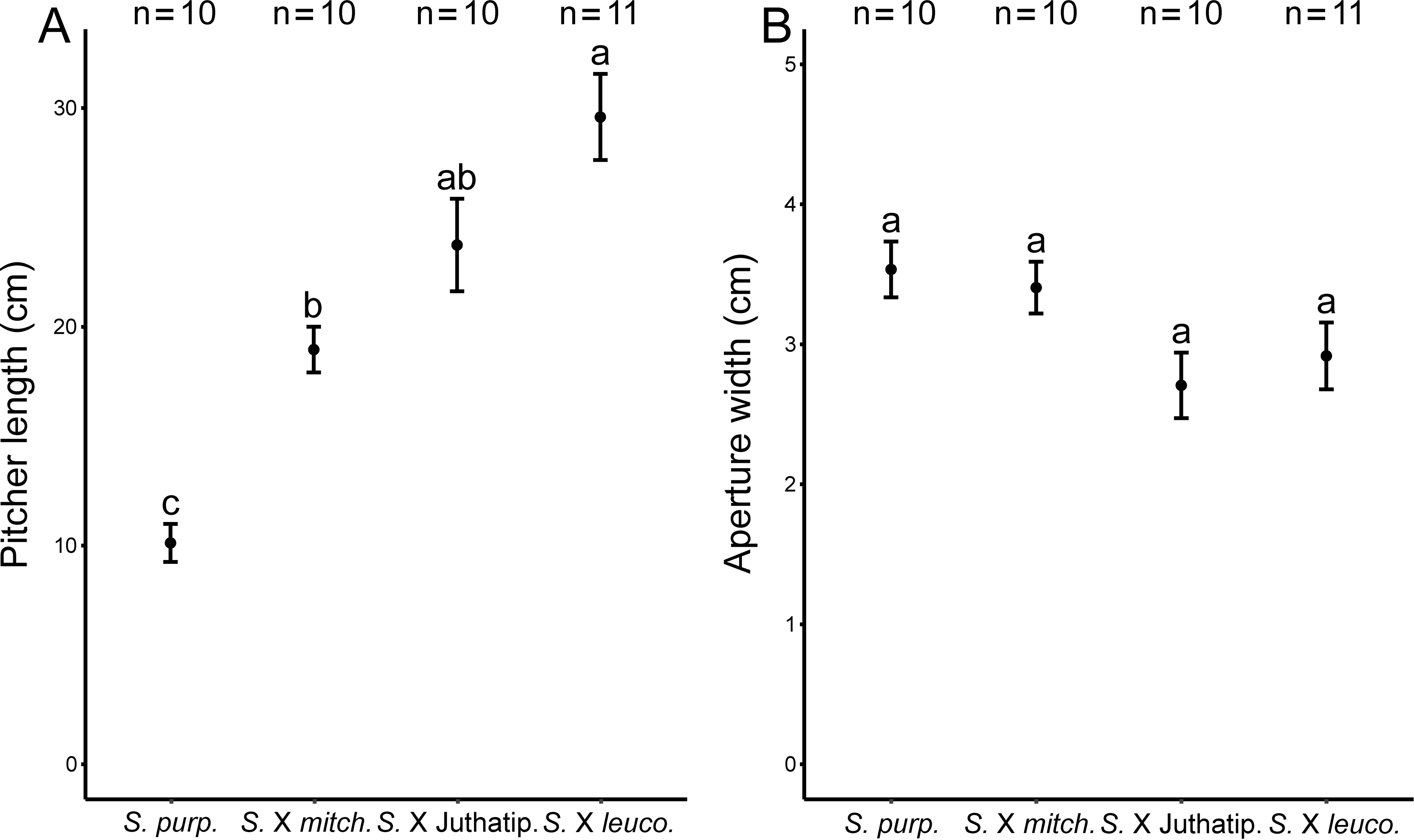

Supplement: S1 Fig — Pitcher length (A) and aperture width (B) in the four Sarracenia taxa. Mean values are presented with their associated standard errors. Different letters show statistically significant differences in means between plant taxa (P<0.05). (TIF) [file pone.0277603.s001.tif]
