# Supplementary material for: Volatile organic compounds influence prey composition in Sarracenia carnivorous plants
Source: PLoS One. 2023 Apr 19;18(4):e0277603. doi: 10.1371/journal.pone.0277603 (PMC10115284; doi:10.1371/journal.pone.0277603)
Supplement: S1 Table — Mean relative amounts (% ± SE) of VOCs in the odour bouquet of the four Sarracenia taxa. VOCs are listed according to their class and calculated Linear Retention Indices (LRI). Mass fragments for unknown compounds were listed with the molecular ion first, followed by the base peak and other fragments in order of decreasing abundance. IA: Identification Attempt, *: Identified with comparison with synthetic standards. Occurrence (Occ.) refers to the proportion of pitchers in which the VOC was found. (PDF) [file pone.0277603.s002.pdf]

			S. purpurea	*S.* X *mitchelliana*	*S.* X Juthatip soper	*S.* X *leucophylla*	
	**Number of individuals sampled**		7	10	9	13	
	**Total number of compounds**		47	56	41	65	
	**Mean quantity of scent emitted (ng/h)**		118.01 (±51.14)	118.63 (±28.42)	45.94 (±20.90)	154.64 (±26.88)	**Occ. (%)**
**Code**	**FATTY ACID DERIVATIVES**	**LRI**					
F1	Oct-1-ene	794	0.18 (±0.16)	0.05 (±0.03)	0.32 (±0.20)	0.22 (±0.17)	56
F2	Heptanal	896	0.05 (±0.05)	0.07 (±0.07)		0.20 (±0.13)	23
F3	Oct-1-en-3-ol*	981	4.06 (±1.88)	1.48 (±1.22)	2.64 (±1.76)	0.61 (±0.33)	51
F4	Octan-2-one	988	0.09 (±0.09)	0.63 (±0.47)			13
F5	Dec-1-ene	991			4.82 (±4.82)		3
F6	Octanal	1006	4.82 (±3.03)	1.48 (±0.53)	1.92 (±1.06)	0.29 (±0.16)	51
F7	Undec-1-ene	1092	1.95 (±1.76)	0.48 (±0.48)	1.91 (±0.83)	0.56 (±0.36)	26
F8	Nonanal*	1108	13.66 (±3.69)	17.77 (±5.90)	7.41 (±3.84)	6.89 (±3.19)	51
F9	Dodec-1-ene	1192		2.44 (±0.72)	1.29 (±0.68)	0.28 (±0.14)	49
F10	Ethyl-octanoate	1196	7.67 (±5.13)	3.45 (±1.89)	5.63 (±3.80)	1.37 (±0.78)	41
F11	Decanal*	1208	8.31 (±4.32)	15.38 (±5.09)	5.41 (±3.16)	1.47 (±0.77)	46
F12	Tridec-1-ene	1289	0.89 (±0.42)	1.03 (±0.41)	3.03 (±0.90)	0.53 (±0.20)	77
F13	Undecanal	1310	0.12 (±0.05)	0.67 (±0.28)	0.14 (±0.10)	0.27 (±0.18)	33
F14	Tetradec-1-ene	1388	0.54 (±0.44)	0.11 (±0.05)	0.09 (±0.06)	0.10 (±0.09)	36
F15	(*Z*)-Jasmone	1402		0.19 (±0.14)	0.32 (±0.32)	1.09 (±0.66)	23
F16	Dodecanal	1406	0.63 (±0.53)	0.31 (±0.15)	0.09 (±0.07)	0.34 (±0.28)	33
	**BENZENOIDS**						
B1	Benzaldehyde*	971		0.11 (±0.11)		0.28 (±0.28)	5
B2	Benzyl alcohol	1042				0.46 (±0.30)	8
B3	Acetophenone	1072	3.51 (±2.06)	1.72 (±1.18)	0.49 (±0.49)	0.45 (±0.45)	18
B4	Methyl benzoate	1102			9.39 (±5.55)	0.37 (±0.37)	15
B5	Phenylethanol*	1121				1.03 (±0.73)	5
B6	Ethyl benzoate	1178			10.86 (±7.29)	2.49 (±1.11)	18
B7	Methyl salicylate*	1202	0.56 (±0.36)			2.34 (±1.23)	23
B8	Isopropyl benzoate	1212				0.03 (±0.03)	3
B9	Isobutyl Benzoate	1337	0.59 (±0.59)	0.21 (±0.14)	0.23 (±0.15)	0.05 (±0.05)	15
	**MONOTERPENOIDS**						
M1	Sabinene	978				0.23 (±0.17)	15
M2	Myrcene*	991	0.75 (±0.29)	0.66 (±0.44)	1.11 (±0.66)	13.45 (±2.95)	56
M3	*p*-Cymene*	1030	15.13 (±6.87)	12.75 (±6.18)	7.33 (±6.31)	12.07 (±6.49)	41
M4	Limonene*	1035	5.41 (±1.59)	3.41 (±1.60)	5.50 (±2.62)	16.81 (±3.13)	74
M5	(*Z*)-*β*-Ocimene*	1036	0.01 (±0.01)		2.18 (±2.17)	0.20 (±0.08)	28
M6	(*E*)-*β*-Ocimene*	1048	3.58 (±1.43)	3.27 (±1.21)	12.14 (±3.31)	20.25 (±3.57)	72
M7	(*Z*)-Linalool oxide (furanoid form)	1076	0.53 (±0.38)	1.48 (±0.61)	0.62 (±0.47)	0.03 (±0.02)	33
M8	(*E*)-Linalool oxide (furanoid form)	1091	4.87 (±2.92)		1.83 (±1.33)		13
M9	Terpinolene	1091				0.24 (±0.17)	5
M10	Linalool*	1102	3.52 (±1.71)	5.96 (±2.03)	3.92 (±2.29)	0.48 (±0.22)	67

M11	allo-Ocimene	1131				0.32 (±0.13)	13
M12	(*E,E*)-Cosmene	1135	0.13 (±0.13)	0.51 (±0.30)	2.18 (±1.76)	0.66 (±0.19)	36
M13	(*E*)-Linalool oxide (pyranoid form)	1179	0.09 (±0.08)	0.16 (±0.05)	0.08 (±0.06)	0.12 (±0.08)	36
	**SESQUITERPENOIDS**						
S1	α-Ylangene	1374		0.34 (±0.20)	0.48 (±0.30)	0.67 (±0.53)	28
S2	α-Santalene	1381	0.18 (±0.09)		0.04 (±0.04)	0.17 (±0.08)	23
S3	Cyclosativene	1385		0.27 (±0.19)		0.05 (±0.05)	10
S4	α-Copaene*	1389	0.26 (±0.24)	0.26 (±0.14)	0.02 (±0.02)	0.12 (±0.06)	33
S5	β-Bourbonene	1398		0.04 (±0.04)		0.03 (±0.03)	8
S6	isoCaryophyllene [IA]	1420	0.05 (±0.05)	0.06 (±0.06)		0.01 (±0.01)	8
S7	(Z)-α-Bergamotene	1423	0.00 (±0.00)	0.02 (±0.02)	0.04 (±0.04)	0.02 (±0.02)	10
S8	β-Ylangene	1434		0.35 (±0.23)		0.12 (±0.10)	10
S9	β-Caryophyllene*	1437	12.28 (±5.47)	13.93 (±5.19)	3.57 (±2.44)	7.51 (±1.85)	79
S10	(E)-β-Bergamotene	1439	0.02 (±0.02)	1.49 (±0.59)	2.13 (±0.81)	0.01 (±0.01)	36
S11	β-Copaene	1442	0.02 (±0.02)	0.20 (±0.12)		0.24 (±0.15)	21
S12	Geranylacetone*	1451	1.85 (±1.62)	0.75 (±0.30)	0.33 (±0.28)	0.03 (±0.03)	41
S13	(E)-β-Farnesene	1456		0.28 (±0.28)		0.09 (±0.09)	5
S14	unknowm Sesquiterpene 1 (91, 105, 161, 119, 81, 204)	1464		0.18 (±0.18)		0.21 (±0.17)	10
S15	Ɛ-Muurolene	1467		0.06 (±0.06)		0.03 (±0.03)	5
S16	α-Humulene*	1473	0.18 (±0.18)	0.14 (±0.14)		0.10 (±0.06)	13
S17	γ-Muurolene	1488		0.42 (±0.30)			5
S18	unknown Sesquiterpene 2 (93, 119, 105, 69, 161, 204)	1493	1.76 (±1.23)	0.07 (±0.07)		1.23 (±0.52)	33
S19	Germacrene D*	1498		1.42 (±0.71)		0.10 (±0.08)	26
S20	γ-Amorphene	1504		0.11 (±0.11)		0.12 (±0.09)	8
S21	(E,E)-α-Farnesene	1507	0.38 (±0.26)	0.05 (±0.05)	0.11 (±0.11)	0.45 (±0.17)	26
S22	β-Curcumene	1509	0.01 (±0.01)	0.10 (±0.07)		0.05 (±0.05)	13
S23	α-Muurolene	1511	0.04 (±0.04)	0.08 (±0.05)	0.01 (±0.01)	0.01 (±0.00)	18
S24	β-Bisabolene	1516	0.02 (±0.02)	0.06 (±0.06)			5
S25	γ-Cadinene	1528	0.15 (±0.15)	0.36 (±0.20)		0.04 (±0.03)	15
S26	δ-Cadinene	1530	0.15 (±0.15)	0.79 (±0.38)		0.03 (±0.03)	23
S27	(Z)-Calamenene	1534	0.14 (±0.14)	0.24 (±0.13)	0.01 (±0.01)	0.02 (±0.02)	15
S28	(E)-Cadina-1,4-diene	1548	0.14 (±0.08)	0.21 (±0.13)	0.05 (±0.05)	0.29 (±0.14)	41
S29	α-Cadinene	1552		0.11 (±0.07)			5
S30	α-Calacorene	1558		1.75 (±0.67)	0.27 (±0.21)	0.30 (±0.22)	28
S31	(E)-Nerolidol*	1566	0.10 (±0.07)	0.09 (±0.07)	0.05 (±0.04)	0.08 (±0.04)	38
S32	Caryophyllene oxide	1575	0.01 (±0.01)			0.01 (±0.01)	5
S33	Hydroxysesquicineole [IA]	1598	0.63 (±0.43)			1.28 (±0.74)	18
