# Supplementary material for: Volatile organic compounds influence prey composition in Sarracenia carnivorous plants
Source: PLoS One. 2023 Apr 19;18(4):e0277603. doi: 10.1371/journal.pone.0277603 (PMC10115284; doi:10.1371/journal.pone.0277603)
Supplement: S2 Table — Results of the pairwise comparisons between taxa associated to the permutational multivariate analyses of variance on the relative amounts of VOCs emitted (A) and prey group trapped (B) by pitchers (Fig 1). P-values were adjusted for multiple comparisons with the Holm’s correction, *: P<0.05, **: P<0.01, and ***: P<0.001. The plant effect (‘Plant identity’ nested within ‘Plant taxon’) was also accounted for. (PDF) [file pone.0277603.s003.pdf]

Dependent variable		A) VOC emitted			(B) Prey trapped		
Pairwise factors	Explanatory variables	F	Df	adjusted P-value	F	Df	adjusted P-value
S. purpurea vs *S.* X *leucophylla*	Plant taxon	5.68	1	0.002 **	6.41	1	0.004 **
	Plant identity	1.56	7	0.050 *	1.29	9	0.242
S. purpurea vs *S.* X Juthatip soper	Plant taxon	1.82	1	0.100	6.16	1	< 0.001 ***
	Plant identity	1.26	5	0.168	1.41	10	0.206
S. purpurea vs *S.* X *mitchelliana*	Plant taxon	1.46	1	0.199	1.28	1	0.299
	Plant identity	3.37	4	0.002 **	1.40	11	0.146
S. X *mitchelliana* vs *S.* X *leucophylla*	Plant taxon	9.26	1	< 0.001 ***	7.13	1	0.002 **
	Plant identity	2.54	7	< 0.001 ***	1.79	12	0.090
S. X *mitchelliana* vs *S.* X Juthatip soper	Plant taxon	2.69	1	0.032 *	6.70	1	0.004 **
	Plant identity	2.24	5	0.005 **	2.58	13	0.016 *
S. X Juthatip soper vs *S.* X *leucophylla*	Plant taxon	3.30	1	0.002 **	2.84	1	0.064
	Plant identity	1.19	8	0.202	1.20	11	0.276
