# Supplementary material for: Volatile organic compounds influence prey composition in Sarracenia carnivorous plants
Source: PLoS One. 2023 Apr 19;18(4):e0277603. doi: 10.1371/journal.pone.0277603 (PMC10115284; doi:10.1371/journal.pone.0277603)
Supplement: S3 Table — Results of the pairwise comparisons between taxa associated to the models carried out to explain the variation in the sqrt-transformed density (number of individuals / pitcher length) of the different insect groups trapped by pitchers. Contrasts were tested between plant taxa using pairwise tests. P-values were adjusted for multiple comparisons with the Holm’s correction. (PDF) [file pone.0277603.s004.pdf]

Dependent variable	Plant taxon	Estimate (±SE)	Df	Contrasts
**Density of Total prey**	*S. purpurea*	0.30 (±0.16)	18.1	b
	S. X *mitchelliana*	0.48 (±0.16)	17.9	b
	S. X Juthatip soper	1.16 (±0.16)	17.9	a
	S. X *leucophylla*	1.16 (±0.16)	15.5	a
**Flying prey**	*S. purpurea*	0.09 (±0.16)	10.6	b
	S. X *mitchelliana*	0.32 (±0.17)	11	b
	S. X Juthatip soper	0.96 (±0.17)	11	a
	S. X *leucophylla*	1.10 (±0.17)	10.2	a
**Ants**	*S. purpurea*	0.22 (±0.12)	23.1	a
	S. X *mitchelliana*	0.29 (±0.12)	27.2	a
	S. X Juthatip soper	0.50 (±0.12)	27.2	a
	S. X *leucophylla*	0.21 (±0.11)	21.2	a
**Bees**	*S. purpurea*	-0.12 (±0.10)	11.4	c
	S. X *mitchelliana*	0.15 (±0.11)	11.8	b
	S. X Juthatip soper	0.77 (±0.11)	11.8	a
	S. X *leucophylla*	0.70 (±0.11)	10.8	a
**Moths**	*S. purpurea*	-0.01 (±0.08)	17.5	c
	S. X *mitchelliana*	0.05 (±0.08)	17.1	c
	S. X Juthatip soper	0.29 (±0.08)	17.1	b
	S. X *leucophylla*	0.48 (±0.08)	14.8	a
**Diptera**	*S. purpurea*	0.03 (±0.12)	11.9	b
	S. X *mitchelliana*	0.14 (±0.12)	12	b
	S. X Juthatip soper	0.37 (±0.12)	12	a
	S. X *leucophylla*	0.52 (±0.11)	10.8	a
**Wasps**	*S. purpurea*	0.08 (±0.08)	20	b
	S. X *mitchelliana*	-0.02 (±0.08)	19.5	b
	S. X Juthatip soper	0.17 (±0.08)	19.5	ab
	S. X *leucophylla*	0.32 (±0.08)	16.6	a
**Beetles**	*S. purpurea*	0.07 (±0.05)	20.7	a
	S. X *mitchelliana*	0.14 (±0.06)	19.7	a
	S. X Juthatip soper	0.13 (±0.06)	19.7	a
	S. X *leucophylla*	0.15 (±0.05)	15.1	a
