# Supplementary material for: Volatile organic compounds influence prey composition in Sarracenia carnivorous plants
Source: PLoS One. 2023 Apr 19;18(4):e0277603. doi: 10.1371/journal.pone.0277603 (PMC10115284; doi:10.1371/journal.pone.0277603)
Supplement: S4 Table — Correlation table relative to odour variables (emission rate of fatty acid derivatives, benzenoids, monoterpenoids and sesquiterpenoids), morphology variables (pitcher length and aperture width) and number of ants, bees, moths, Diptera, wasps and beetles trapped in pitchers of S. X leucophylla. The correlation coefficients between each pair of variables estimated using the Pearson method, are presented with their associated P-values, *: P<0.05, **: P<0.01, and ***: P<0.001. (PDF) [file pone.0277603.s005.pdf]

**Variables**	Fatty. emission rate	Benz. emission rate	Mono. emission rate	Sesqui. emission rate	Pitcher length	Aperture width
Benzenoids emission rate	0.02					
Monoterpenoids emission rate	-0.04	0.78*				
Sesquiterpenoids emission rate	-0.20	0.86**	0.57			
Pitcher length	-0.43	0.45	0.58	0.52		
Aperture width	-0.29	0.69*	0.67*	0.66	0.92***	
Number of ants	0.69*	-0.22	-0.28	-0.32	-0.78*	-0.71*
Number of bees	-0.17	0.84**	0.80**	0.86**	0.66	0.75*
Number of Diptera	-0.26	0.75*	0.86**	0.53	0.51	0.67*
Number of moths	-0.17	0.91***	0.85**	0.89**	0.72*	0.85**
Number of wasps	-0.18	0.67*	0.77*	0.35	0.40	0.52
Number of beetles	-0.37	0.18	0.11	0.49	0.39	0.27
