# Supplementary material for: Volatile organic compounds influence prey composition in Sarracenia carnivorous plants
Source: PLoS One. 2023 Apr 19;18(4):e0277603. doi: 10.1371/journal.pone.0277603 (PMC10115284; doi:10.1371/journal.pone.0277603)
Supplement: S5 Table — Effects of the variables retained in the second-best Poisson multiple regression model to explain variation in the number of ants, bees, moths, Diptera, wasps and beetles trapped in pitchers of S. X leucophylla. (PDF) [file pone.0277603.s006.pdf]

Dependent variable	Explanatory variables	Estimate (±S.E.)	P-value	AIC	Null model AIC
**Number of ants**	Intercept	3.77 (±1.36)	0.0058 **	30.2	45.6
	Pitcher length	-0.15 (±0.07)	0.0188 *		
	Fatty acid derivative quantity	0.05 (±0.02)	0.0372 *		
	Benzenoid quantity	0.02 (±0.02)	0.2790		
**Number of bees**	Intercept	-1.45 (±0.99)	0.1453	55.8	101.1
	Pitcher length	0.09 (±0.03)	0.0026 **		
	Monoterpene quantity	0.01 (±0.002)	<0.001 ***		
	Fatty acid derivative quantity	0.01 (±0.01)	0.2762		
**Number of moths**	Intercept	-3.37 (±1.55)	0.0296 *	39.2	69.7
	Pitcher length	0.13 (±0.05)	0.0054 **		
	Monoterpene quantity	0.01 (±0.002)	0.0024 **		
	Fatty acid derivative quantity	0.02 (±0.02)	0.2075		
**Number of diptera**	Intercept	0.47 (±0.37)	0.1960	57.4	127.8
	Monoterpene quantity	0.01 (±0.002)	<0.001 ***		
	Fatty acid derivative quantity	-0.02 (±0.01)	0.1050		
**Number of wasps**	Intercept	-0.87 (±0.65)	0.1850	42.4	60.6
	Monoterpene quantity	0.02 (±0.004)	<0.001 ***		
	Sesquiterpene quantity	-0.01 (±0.01)	0.1810		
**Number of beetles**	Intercept	-1.46 (±2.12)	0.4891	36.4	38.0
	Pitcher length	0.07 (±0.06)	0.2580		
	Fatty acid derivative quantity	-0.05 (±0.05)	0.2950		
